# Sonochemically Fabricated Microelectrode Arrays for Use as Sensing Platforms

**DOI:** 10.3390/s100505090

**Published:** 2010-05-24

**Authors:** Stuart D. Collyer, Frank Davis, Séamus P.J. Higson

**Affiliations:** 1 Microarray Ltd, PO BOX 88, Manchester, M60 1QD, UK; E-Mail: s.d.collyer@cranfield.ac.uk; 2 Cranfield Health, Cranfield University, Cranfield, Bedfordshire, MK43 0AL, UK; E-Mail: f.davis@cranfield.ac.uk

**Keywords:** microelectrodes, arrays, fabrication, surface modification, enzymes

## Abstract

The development, manufacture, modification and subsequent utilisation of sonochemically-formed microelectrode arrays is described for a range of applications. Initial fabrication of the sensing platform utilises ultrasonic ablation of electrochemically insulating polymers deposited upon conductive carbon substrates, forming an array of up to 70,000 microelectrode pores cm^−2^. Electrochemical and optical analyses using these arrays, their enhanced signal response and stir-independence area are all discussed. The growth of conducting polymeric “mushroom” protrusion arrays with entrapped biological entities, thereby forming biosensors is detailed. The simplicity and inexpensiveness of this approach, lending itself ideally to mass fabrication coupled with unrivalled sensitivity and stir independence makes commercial viability of this process a reality. Application of microelectrode arrays as functional components within sensors include devices for detection of chlorine, glucose, ethanol and pesticides. Immunosensors based on microelectrode arrays are described within this monograph for antigens associated with prostate cancer and transient ischemic attacks (strokes).

## 1. Introduction

It is well understood that the total rate of mass transport to an electrode surface depends on the bulk concentration of analyte, the area of the electrode, as well as diffusional and convectional conditions. Although the use of diffusion-limiting membranes can help limit the rate of mass transport, in practical situations it may be preferable to increase the rate of mass transport. This result can be obtained by using microelectrodes [1]. Although the advantages offered by very small electrodes have been recognised by physiologists for many years [2,3], research in this area did not become very active until the work of Fleischmann and co-workers [4–6] continued with advances in the field of electronics. Their work especially dealt with the measurement of very small currents where the advent of microstructural materials provided the tools necessary to develop the use of microelectrodes in practical applications [7].

**Figure 1.**
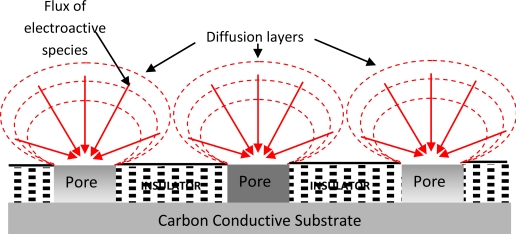
Schematic representation of diffusion at a microelectrode array.

The electrochemical responses at microelectrodes can differ greatly from those seen at conventional (*i.e.*, macro) electrodes. Typically, microelectrodes offer higher sensitivity, smaller double-layer capacitance, and lower Ohmic losses that result in higher signal-to-noise ratios. A detailed review into the enhancing properties of microelectrodes and microelectrode arrays has been published elsewhere [8]. The improved diffusion characteristics of microelectrodes allow measurements in both static and stirred solutions [9]. This characteristic has opened many new possibilities in electrochemistry and may have a positive impact on the commercial realisation of chemical sensors since it allows a sensor to be used as an “insertion” device. Such sensors may be dipped into the analyte solution without stirring effects (convection), which can cause unwanted fluctuations in the electrode response. To date, the majority of sensors which have reached commercial success have avoided this problem by inhibiting stirring by some means (e.g., droplets of blood in hand-held glucose sensors are held by surface tension); however, this is not possible for all applications, such as the analysis of non-viscous media or the analysis of analytes present in flowing streams. Microelectrodes have several properties which make them attractive as active elements within sensors for the determination of analytes in, for example, flowing water streams. Specifically, microelectrodes exhibit enhanced diffusion in comparison to larger sensors and this leads to enhanced sensitivity—as well as independence from the effects of convectional mass transport and therefore solution flow or other movement. Individual microelectrodes offer however very small responses and one approach for overcoming this problem is via the use of many microelectrodes coupled together in the form of an array to allow a larger cumulative response to be measured, as depicted in Figure 1.

Despite this no microelectrode-based array sensors have undergone successful widespread commercialisation, largely due to the cost of conventional fabrication routes such as photolithography or laser ablation. A recent review of the design and fabrication of single microelectrodes has been given by Zoski [10]. The most popular materials include platinum, carbon fibres, and gold, although mercury, iridium, nickel, silver, and superconducting ceramics have also been used. Microdisc electrodes predominate because of their relative ease of construction, and the ability to polish the sensing surface. They are commonly fabricated by embedding a platinum wire or carbon fibre into tapered glass pipettes that are subsequently sealed with epoxy resin. An active electrode surface is then exposed by mechanical polishing. An alternative procedure involves the electropolymerisation of a passivating polymer film around the carbon fibre electrode. Both of these methods give electrodes with total diameters in the tens-of-micrometres range. They are however more expensive to fabricate than large electrodes and are therefore often restricted to use within research applications and *in vivo* analysis (such as dopamine monitoring in the mammalian brain).

Multiple microelectrode arrays for electromechanical sensors have been fabricated via photolithographic or laser techniques in a similar manner to the production of printed circuit board and silicon micro-chips or laser ablation, since the late 1970s [11]. Other array fabrication methods include immobilisation of large numbers of metallic sites within a non-conducting support and the electrodeposition of mercury and platinum within the pores of a polymer membrane.

With respect to carbon, microelectrodes have been fabricated using conventional techniques such as the suspension of carbon particles or fibres in an insulating matrix, the impregnation of porous carbon within an insulator, or the impregnation of the pores of a host membrane with conducting carbon particles [12]. Furthermore, glassy carbon electrodes [13–15], reticulated vitreous carbon [16] and pyrolytic carbon [17,18] arrays have also received considerable attention. Electrodes that are constructed by conventional techniques are subject to great variability in their physical, electrical, and electrochemical characteristics. Furthermore, these electrodes are difficult to reproducibly fabricate in multi-electrode arrays, and such arrays, when realised, can contain appreciable capacitative coupling between electrodes [19].

Different types of carbon array electrodes have also been fabricated using photolithographic techniques [20,21], including microdiscs [22] and inter-digitated array electrodes [17,23]. Although such photolithographically produced arrays of microelectrodes that exhibit reduced capacitative coupling can be fabricated reproducibly—they may incur very high costs and are therefore not commercially viable for mass production, especially if disposable tips are required, which is often the case.

In spite of the inherent advantages of microelectrodes previously mentioned, their small size does present some drawbacks. Given that signal size is dependent upon electrode area, it follows that a microelectrode will only generate a very small current (typically of the order of picoamps). This places a serious demand on the instrumentation equipment used to prevent the signal being swamped by electrical noise. However, it is possible to circumvent the problem of a small signal if multiple microelectrodes in the same system are connected in parallel. The result is that a significant current can be obtained, whilst retaining the inherent performance advantages of a single microelectrode. Such assemblies are termed microelectrode arrays [24].

Particularly due to high manufacturing costs, microelectrode arrays have yet to be commercially realised in ‘one-shot’ sensor applications and consequently, the advantageous properties that they bring with them are also yet to be seen in disposable sensor type applications. However, a new novel method for microelectrode array fabrication that lends itself perfectly to mass production, due to the simplicity and inexpensiveness of the approach, has been developed within our laboratories. The rationale underpinning this work is that sonochemical ablation of thin insulating polymer films upon electrode surfaces may expose defined areas, each of which can act as localised microelectrodes and thus collectively as a microelectrode array.

## 2. Fabrication of Microelectrode Arrays

**Figure 2.**
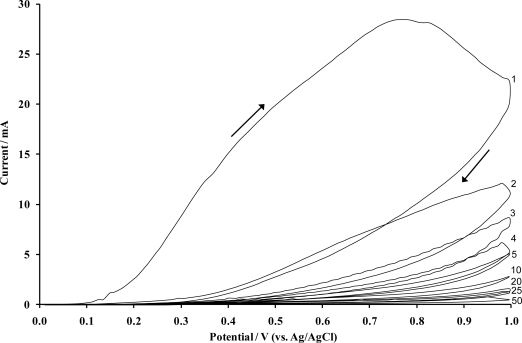
Cyclic voltammogram obtained following the electropolymerisation of 5 mM o-phenylenediamine dihydrochloride on a sheet of 100 screen printed carbon electrodes (50 Sweeps). **[**Supporting electrolyte: phosphate buffer (pH 7.4, 5.1 × 10^−3^ M). Scan rate: 50 mVs^−1^].

Electropolymerisation of heteroaromatic compounds on electrode surfaces has become one of the many effective methods used to modify electrodes. Among electrodeposited polymers, the material obtained by the voltammetric electropolymerisation of *o*-phenylenediamine (*o*PD), commonly known as poly(*o*-phenylenediamine) (P*o*PDA) or poly(diaminobenzene) in particular lends itself to this application since it forms strongly adherent, highly reproducible membranes [25], which completely cover the electrode surface regardless of dimension. The use of such films not only provides an economic advantage, but the electropolymerisation of *o*PD from solution yields non-conducting and electro-inactive P*o*PDA films. Such synthesis also offers possibilities of producing self regulating, ultra-thin and essentially defect free films with uniform thickness, since they only grow thick enough to become insulators [26]. The formation of ultra-thin insulating layers of P*o*PDA via the electrochemically initiated polymerisation of *o*PD is performed upon the surface of carbon supporting electrodes. P*o*PDA can be formed electrochemically using a constant potential or cyclic voltammetric method, however, previous work has shown that the use of cyclic voltammetry results in a more useful film for use within sensors in terms of enhanced resistance to interferents [27]. A resultant voltammogram, depicting the polymerisation process, is shown in Figure 2.

**Figure 3.**
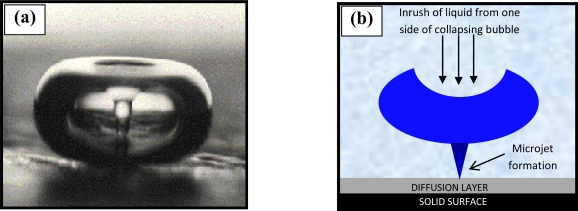
**(a)** A flash microphotograph (Crum [32]—Figure reproduced with permission ©1982 IEEE) and **(b)** a schematic representation, of a microjet of liquid streaming through a bubble upon implosion due to cavitation near a solid surface.

The voltammogram is defined by a large anodic irreversible oxidation peak on the first scan, which is characteristic of almost all aromatic diamines and its position is believed to be specific to each differing monomer [28]. The large anodic peak is then followed by significantly smaller and decreasing anodic waves in successive scans (Figure 2), hence, the electrode becomes progressively insulated by the polymer film as it is deposited on the electrode surface. The anodic irreversible peak current decreased to nearly the background level on the 20th cycle, indicating that the electrode has become totally insulated and therefore cannot be coated with any further polymer. Thus, it can be reasonably assumed that the PPD film is “defect” free, and therefore insulating. The insulated surface is then exposed to an ultrasonic ablation, which leads to the formation of the microelectrode array. Ultrasound (in the kHz range) passing through a solvent such as water causes thermal agitation and localised hotspots of up to several hundred to a few thousand K, which in turn gives rise to the formation of superheated vapour bubbles [29,30]. These bubbles are, however, cooled by the solvent at ambient temperature (in our studies 25 °C) and asymmetrically implode with the ejection of micro-jets of solvent at speeds of up to several hundred ms^−1^ [31]. A schematic of the approach is shown in Figure 3.

This cavitation is known as transient (inertial) since transient cavities generally exist for no more than a few acoustic cycles during which time they expand to at least double their initial radius before collapsing violently within a few microseconds. These micro-jets may cause the shattering of hard brittle solids and this is exploited, for example, within medicine for the shattering of kidney stones. Soft solid surfaces such as polymers may, however, be ablated by such jets [29]. This ablation technique is used to “drill” holes in this insulating material, through to the underlying carbon substrate, with pore sizes of 0.1 to several microns and a density of up to 70,000 pores cm^−2^. Optimum fabrication conditions have been elucidated via studies undertaken within our laboratories, and have shown that an ablation time of 20 seconds at 25 kHz produces the most efficient microelectrode arrays in terms of both signal strength and stir-independent behaviour. For brevity here, a summation of these studies can be found elsewhere [33].

**Figure 4.**
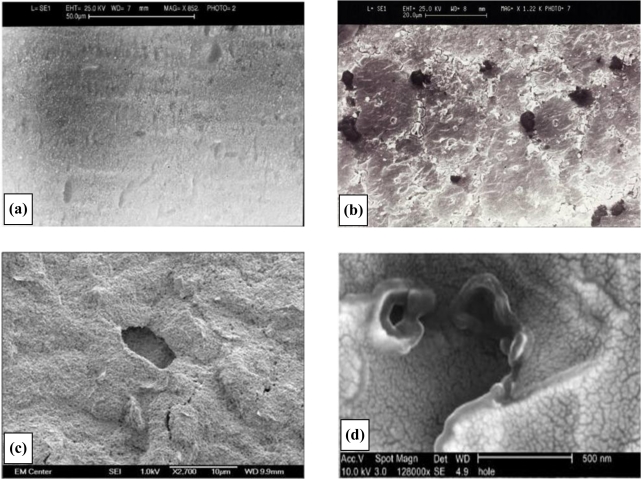
Deposition of poly(diaminobenzene) to form an insulating film. **(a)** Electron micrograph of insulated poly(diaminobenzene) insulated electrode surface. **(b)** Electron micrograph of sonochemically fabricated microelectrode array. **(c)** Close-up of sonochemically fabricated microelectrode array showing pore structure. **(d)** Enhanced magnification showing polymer “peel” at microelectrode pore.

Scanning electron micrographs for the various fabrication stages of a 20 second sonicated electrode assembly (ablated at 25 kHz) are shown in Figure 4. There are several features of interest within these micrographs. Firstly the distribution of the pores is random since ultra-sonic cavitation is a chaotic process. It is also evident that almost all of the cavities are bimodal in size, possessing either ∼3 μm (±1 μm) or sub-micron diameters. Microelectrodes are defined as electrodes with diameters of tens of microns or less [8], and our pores easily fit into this category. We believe that the smallest of the cavities observed are formed by the initial impact of the micro-jets of fluid [29]. These cavities are known to act as nucleation sites for further bubble formation [29] and it is thought that the cavity grows as new bubbles implode within the confines of the original cavity. This process gives rise to a quantum enlargement in the diameter of a cavity. Since no larger pores are seen it is believed that the 3 μm diameter pores no longer act as nucleation sites. In some instances there is evidence of a few pores joining to form dumbbell shaped cavities when two pores form in close proximity to each other, Figure 4b. Figure 4c and 4d shows progressive magnifications of a pore site, and it is noticeable in Figure 4d how the polymer coating has been peeled back from the substrate during the ablation process. These images show the random formation of the microelectrode arrays, and it is worth noting here that this process offers, in essence, not the placement of *regularly-spaced* arrays of microelectrodes but the cumulative effect of a many microelectrodes, all interlinked via the underlying conductive substrate, thus forming an array.

The process can be scaled up to allow for increased manufacture of microelectrode array-based probes. As the polymerisation of P*o*PDA is self limiting a large number of inter-connected electrodes can be polymerised simultaneously. The sensors produced offer all the advantages that are associated with microelectrodes (and the subsequent arrays):

1. The ability to work in highly resistive solutions without adding any supporting electrolyte (reduced effects of solution resistance).
2. A dramatic increase of the signal/noise ratio (reduced effects of double layer capacitance).
3. Access to fast kinetics measurements using high speed (thousands V s^−1^) cyclic voltammetry.
4. Negligible consumption of electroactive species during an electroactive redox process at the electrode surface.
5. Establishment of steady-state behaviour, even in static conditions.
6. Low incidence of the solution flow at the electrode surface (*i.e.*, stirring), on the measured current.

### 2.1. Electrochemical Assessment of Microelectrode Arrays

**Figure 5.**
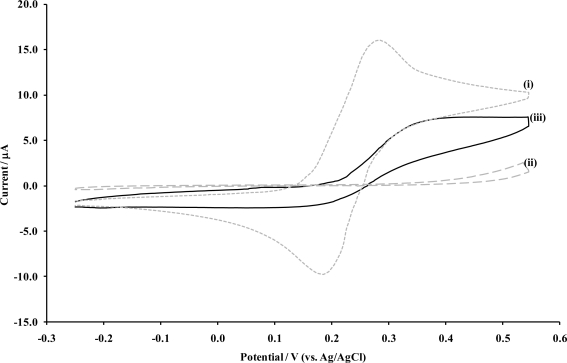
Cyclic voltammetry of 1 mM ferrocenemonocarboxylic acid, at **(i)** a bare carbon electrode, **(ii)** a P*o*PDA coated carbon electrode and **(iii)** a similar electrode subsequently sonicated for 20 seconds. [Supporting electrolyte: phosphate buffer (0.1 M, pH 7.4). Scan rate: 20 mVs^−1^].

Microelectrodes are known to exhibit sigmoidal shaped cyclic voltammograms for reversible solution bound redox species, a characteristic feature that results from the development of radial diffusion at each microelectrode surface, as the hemispherical diffusion profile of microelectrodes allows for a substantially increased flux of electroactive species to the electrode. This is in contrast with the characteristic peak shaped voltammograms expected at a planar, macro-electrode surface, associated with linear diffusion. This phenomenon can therefore be used as a diagnostic tool for assessment of the fabrication of microelectrodes arrays under discussion here, and is depicted in Figure 5.

One of the most significant advantages of microelectrodes is that the steady-state current, which is reached extremely quickly after the application of the potential step, is essentially convection independent. As the diffusion layer expands into solution, it adopts a hemispherical shape, with a gradually increasing surface area and thus an increasing “catchment” area for reagent. Thus, under these conditions, diffusion is faster than convection, and therefore convection effects will not influence the steady-state current response. This is the advantage of the use of microelectrodes in flowing systems in comparison to larger macro-electrodes.

**Figure 6.**
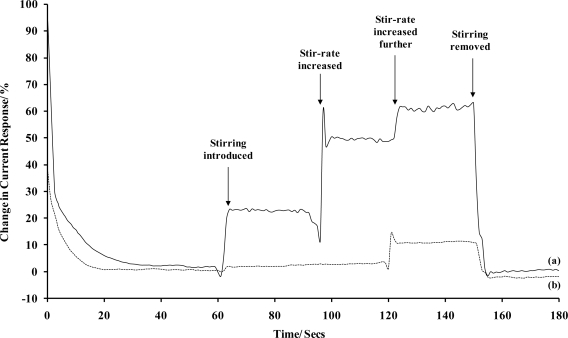
Stir-rate dependence in terms of percentage current change for **(a)** a bare carbon electrode and **(b)** a sonochemically fabricated microelectrode array (average response of 10 sensors), in 1 mM hexaammineruthenium(III) chloride. [Supporting electrolyte: phosphate buffer (0.1 M, pH 7.4). Polarising potential: −0.35 V (*versus* Ag/AgCl)].

Sonochemically fabricated carbon microarrays were therefore polarised in 1 mM hexaammineruthenium(III) chloride/buffer solutions for 180 seconds and compared to planar carbon electrodes in terms of relative percentage change in current due to convection. It can be seen that the planar electrode is clearly influenced by convection (Figure 6). Contrastingly, the sonochemically fabricated microarray exhibit little change in current when stirring is introduced to the analyte sample. This direct comparison clearly indicates the major advantage that such arrays can have over planar electrodes when applied to sensor systems. Convection has no influence on the current for two (significant) levels of convection and even at the very highest stir-rate, only a 10% change in current occurs at the microarrays compared with 65% at the planar electrodes.

### 2.2. Surface Modification of Microelectrode Arrays

**Figure 7.**
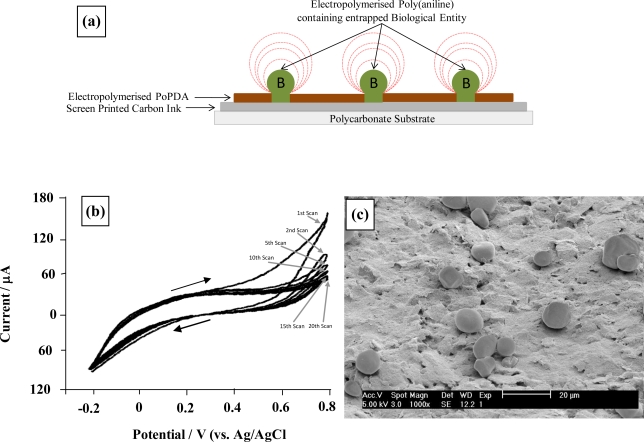
Generation of enzyme containing-poly(aniline) protrusion microelectrode arrays. **(a)** Schematic of sonochemically fabricated poly(aniline)/microelectrode array; **(b)** cyclic voltammetry (50 mV s^−1^) for electropolymerisation of poly(aniline) and co-entrapment of glucose oxidase; **(c)** Scanning electron micrograph of sonochemically fabricated poly(aniline) enzyme microelectrode array.

These conductive microelectrodes arrays can be utilised as base substrates for the deposition of further recognition layers. A method developed within our research group involves the utilisation of a second electropolymerisation step to grow “mushroom-like” protrusions of a conductive polymer (such as poly(aniline)) from these pores. Scanning electron micrographs (Figure 7) clearly show formation of these protrusions—and this allows species capable of chemical or biochemical recognition to be incorporated directly within the conducting polymer. As can be seen these protrusions can be easily grown to at least 10 microns in diameter, the size of these being controlled by the number of deposition cycles. It is worth noting here that all of the microelectrode cavitations appear to be filled with poly(aniline), including the very smallest of the ∼0.1 μm diameter pores. This shows all ablative cavitations of the insulating polymer film lead to exposure of the underlying conductive surface, since aniline could not be electropolymerised without access to the underlying conducting surface.

## 3. Application of Microelectrode Arrays within Sensing Systems

### 3.1. Chlorine Sensor

Chlorine is a powerful oxidising agent that is used widely as a disinfectant in the treatment of industrial, recreational and potable drinking water. A variety of industrial processes are heavily dependant on the use of chlorine because of its potency as a sterilising agent for water, and it is essential that individual users and companies are able to measure chlorine concentrations to determine if adequate levels for disinfection are present. However, in addition to its benefits, the use of chlorine does incur some disadvantages and the presence of excessive concentrations of chlorine can in some cases be detrimental to human health and aquatic life. Concern over the environmental and health effects of chlorination have led to a raft of legislation relating to its determination (viz: European Economic Community Directive 80/778/EEC, 1998; US Environmental Protection Agency, 2000). It is clear that accurate, sensitive and simple procedures are required for the monitoring of chlorine by users and regulatory agents in order to assure compliance with regulations. The majority of chlorine testing within water is performed by colourimetric wet chemistry approaches and a wide range of commercial tests are available for different applications. These however suffer from a number of limitations including bleaching of the colour and a limited analytical range due to fluctuations in colour change. Colourimetric approaches also require skilled operators for use, the equipment is cumbersome and sampling/testing is time consuming [34]. A technique which would permit easy, rapid, accurate and qualitative analysis of chlorine for both free and total determination over a wide analytical range would clearly be advantageous.

The microelectrode pore array could be utilised within an electrochemical sensor for chlorine. Chlorine in aqueous solution can exist as free chlorine (Cl_2_) or in a variety of combined forms such as hypochlorous acid (HOCl) or other forms upon reaction with organic materials. Ammonia will for example react with chlorine to give NH_2_Cl.

Experimental electrodes are based on commercial screen printed electrodes supplied by Microarray Ltd (Manchester) and contain two working electrode microarrays; one dedicated to the measurement of free chlorine and the other for measuring total chlorine. The electrochemical determination of chlorine is based on known titrimetric approaches. Chlorine (both free and combined) reacts with iodide ion to produce iodine which can then be titrated using sodium thiosulphate. Free chlorine can be directly detected electrochemically whereas combined chlorine will not directly reduce at an electrode surface and so acidified potassium iodide is added to the solution, preferentially oxidising both free chorine and combined chlorine according to Equations 1a and 1b.

(1a) $$2I^{-}+{\text{Cl}}_{2}\to I_{2}+2{\text{Cl}}^{-}$$

(1b) $$2H^{+}+{\text{NH}}_{2}\text{Cl}+2I^{-}\to {\text{NH}}_{4}^{+}{\text{Cl}}^{-}+I_{2}$$

The iodine generated can be reduced at the working electrode to produce iodide, according to Equation 2, thereby allowing electrochemical detection.

(2) $$I_{2}+2e^{-}\to 2I^{-}$$

**Figure 8.**
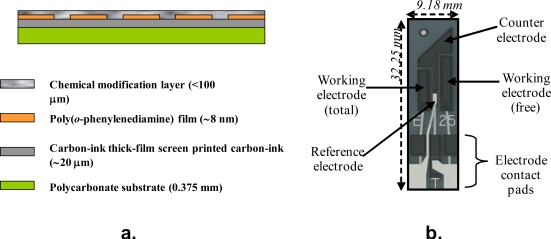
**(a)** Schematic of final sensor construction; **(b)** final four-electrode assembly.

Provided sufficient iodine is made available initially, the current measured will be proportional to the concentration of total residual chlorine in the mixture. For this reason the microelectrode arrays were coated with a thin film of potassium iodide containing formulation using a BioDot AD 3200 dispensing platform, incorporating a BioJet Plus^™^ 3000 dispensing system. These electrodes were then used to measure free and total chlorine respectively. Subtraction of the free chlorine value from the total chlorine level gives the level of combined chlorine. A schematic of the final sensor construction is displayed in Figure 8a, along with a photograph of a “base” electrode (Figure 8b).

Although when placed in aqueous sample solutions, the iodide will dissolve, diffusion away from the electrode is relatively slow and a result for chlorine can be obtained with our system in 30 seconds, long before loss of iodide becomes a problem. Since these electrode assemblies have been developed for single-use only, it does not matter if the iodide is removed during the first measurement.

**Figure 9.**
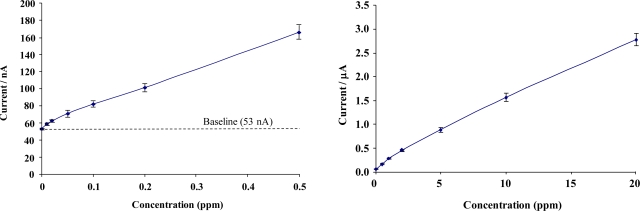
Total chlorine calibration curve; “Low” range from 0 to 0.5 ppm (Mean RSD 4.1%), “High” range from 0 to 20 ppm (Mean RSD 4.3%).

Figure 9 shows calibration profiles obtained for total chlorine solutions interrogated with chemically modified microelectrode array sensors (concentrations ranging from 0 to 20 ppm). Given the range of the current responses obtained, data is presented in low- and high-range calibration curves. Error bars represent the relative standard deviation (RSD) from the 5 sensors taken at each chlorine concentration. The full sensor calibration data exhibits a quasi-linear response to total chlorine solutions, resulting in diminishing escalation in response with increasing concentration.

**Figure 10.**
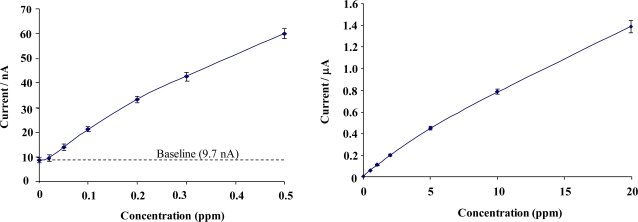
Free chlorine calibration curve; “Low” range 0 to 0.5 ppm (Mean RSD 3.8%), “High” range free chlorine calibration curve from 0 to 20 ppm (Mean RSD 4.2%).

A response of approximately 3 nA per 0.01 ppm for low range total chlorine and 1 nA per 0.01 ppm for high range concentrations was obtained – sufficiently large to permit the differentiation of 0.01 ppm total chlorine. Figure 10 demonstrates similar calibration profiles for concentrations of 0–20 ppm free chlorine interrogated with modified screen printed carbon-ink based microelectrode array sensors. Once again, a quasi-linear calibration profile is obtained for the BioDot modified microelectrode arrays sensors with a response of approximately 2 nA per 0.02 ppm increment for low range free chlorine and 1 nA per 0.02 ppm for high range concentrations.

As expected, there is a considerably smaller amperometric response for free chlorine when compared to the total chlorine response. This process has proved suitable for scaling up for commercial production. Large sheets of screen-printed sensors containing hundreds of individual four electrode (two working, a counter and an Ag/AgCl reference) units can be easily constructed by industrial partners. Insulating layers can be simultaneously deposited onto all the working electrodes and the use of large sonic tanks allows the ablation of the electrode sheets. Thousands of chlorine sensors could be produced daily within our pilot scale facility.

Within this section, we have applied fabricated microelectrode arrays to the determination of chlorine, incorporating a method for localising the chemical reagents required for chlorine detection at the microelectrode array surface. Free, total (and thus combined) forms of chlorine were detected in solutions in the range of 0–20 ppm. Microelectrode array responses for both free and total chlorine analysis were found to show a measureable resolution of down to 0.02 ppm and 0.01 ppm respectively using chronoamperometric methods, well within the required limits defined under current legalisation. Particularly, the microelectrode arrays offer good signal differentiation at the low levels of chlorine concentration, for both forms, afforded by the dramatic increase of the signal/noise ratio that microelectrodes offer over conventional macroelectrodes.

### 3.2. Enzyme-Based Sensors

Many other workers have studied the immobilisation of enzymes (e.g., the oxidases and dehydrogenases) within conducting polymers such as poly(aniline) or poly(pyrrole) for use within sensors [35,36]. We have adapted these initial studies to produce a technique that allows the co-deposition of an enzyme within the conducting polymer, poly(aniline), at conducting microelectrode cavities to form conductive protrusions. As mentioned previously, this modification procedure makes use of the initial formation of microelectrode array pores, from which the poly(aniline) can be “grown”. Addition of a suitable enzyme into the aniline monomer, prior to electro-polymerisation, allows for the production of enzyme sensors, based upon microelectrode technology.

#### 3.2.1. Glucose Oxidase

Microelectrode arrays were fabricated by ablating P*o*PDA films (formed as shown in Figure 2) for 20 seconds (at 25 kHz). For the polymerisation of glucose oxidase enzyme microelectrode arrays, a pH 4.5 phthalate buffer was first prepared using 0.2 M potassium hydrogen phthalate and 0.2 M NaCl. A 0.2 M aniline solution was then prepared in the phthalate buffer. Glucose oxidase, (500 units mL^−1^) was prepared in distilled water, to avoid denaturing the enzyme. 2.7 mL of the buffer and enzyme preparation (1.35 mL each) were mixed in a small volume PTFE cell, immediately prior to the electrochemical coating procedure being performed. Aniline/GOD was polymerised by potentially sequentially cycling for 5 min between −200 and +800 mV *versus* Ag/AgCl at 50 mVs^−1^, film growths were terminated at −0.2 V. Immediately following polymerisation, the working electrode was submerged in pH 7.4 phosphate buffer to minimise enzyme denaturisation. Enzymic microelectrode arrays of this type may be interrogated by both (i) amperometric and (ii) impedimetric approaches. Enzyme electrodes were polarised at +650 mV versus Ag/AgCl for the interrogation of glucose responses as a first demonstration of the enzymic microelectrode array. Glucose oxidase catalyses the production of H_2_O_2_ in the presence of both glucose and oxygen, Equation 3. This approach allows for the interrogation of the sensor based on the amperometric monitoring of H_2_O_2_, Equation 4.

(3) $$\begin{array}{ccc} \text{Glucose}+O_{2} & \overset{\text{Glucose}\;\text{Oxidase}}{\to } & \text{Gluconolactone}+H_{2}O_{2} \end{array}$$

(4) $$\begin{array}{ccc} H_{2}O_{2} & \overset{+650\text{mV}(\text{vs}.\text{Ag}/\text{AgCl})}{\to } & 2H^{+}+O_{2}+2e^{-} \end{array}$$

**Figure 11.**
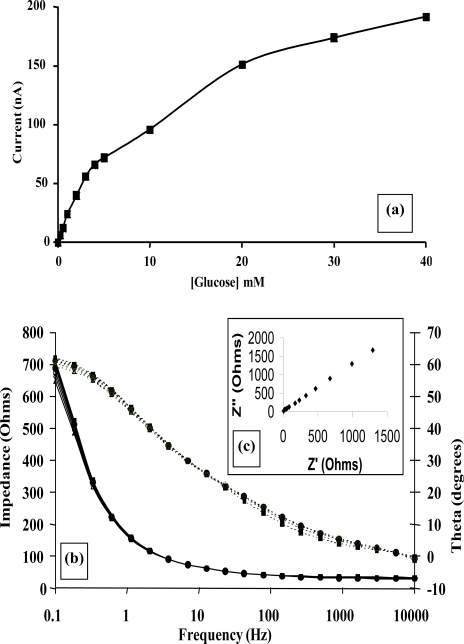

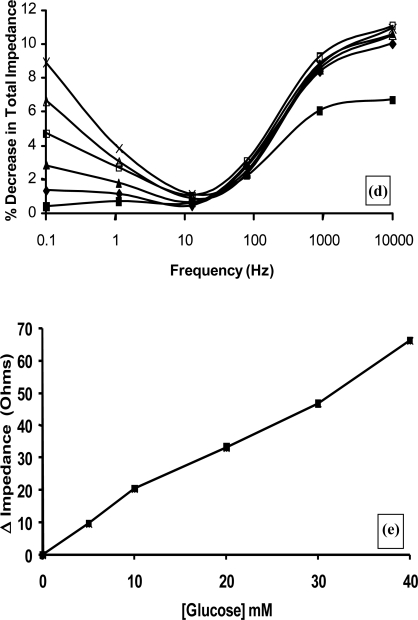
**(a)** Amperometric glucose calibration curve for poly(aniline)/glucose oxidase microelectrode array. **(b)** Total impedance (solid line) and phase angle (dashed line) of microelectrode arrays immersed in glucose/phosphate buffer solutions. Concentration of glucose: 0 mM (■), 1 mM (♦), 5 mM (▴), 10 mM (*), 20 mM (□), 30 mM (▵), 40 mM (×)—N.B. Plots are seen to converge due to scale utilised. **(c)** Complex plane plot for a microelectrode array in 20 mM glucose. **(d)** Percentage change in impedance for microelectrode arrays in various concentrations of glucose *versus* phosphate buffer. Concentration of glucose: 1 mM (■), 5 mM (♦), 10 mM (▴), 20 mM (□), 30 mM (▵), 40 mM (×). **(e)** Calibration plot of impedance change *versus* glucose concentration for a 20 seconds sonicated GOD microelectrode.

The calibration curve of Figure 11a, clearly demonstrates that an amperometric enzymatic sensor may be produced (with stir-independent responses) that could be exploited for a variety of applications in which various flow conditions may be encountered.

Impedimetric measurements for enzyme electrode sensors were recorded in the plane of the working microelectrode array→analyte→solution→electrode. AC impedance spectra were first recorded for glucose oxidase/poly(aniline) sonochemically fabricated microelectrode arrays across a range of frequencies from 0.1 through to 10,000 Hz upon ±5 mV ac excitation (0 V bias) [37], in order to characterise electrochemical redox behaviour and sensor performance for a range of differing concentrations of glucose within O_2_ saturated conditions, Figure 11b. This bias potential allows monitoring of the impedance (conductivity) of the polymer without interference from, for example, the oxidation of H_2_O_2_ or biological interferents such as ascorbate. It is clear that AC Bode impedance spectra show a clear trend towards lowered impedances upon increasing concentration of glucose, with lowered impedances also occurring at higher frequencies.

Control experiments were also performed by recording impedance spectra for enzyme free poly(aniline) microelectrode arrays (not shown for brevity) and showed minimal impedance changes to differing concentrations of glucose, confirming that the sensor responses observed within Figure 11b were indeed enzymatic in nature [33]. It is firstly clear that increasing concentrations of glucose give rise to lowered impedances, via the enzymic activity of glucose oxidase. A plot of total impedance *versus* frequency is shown in Figure 11b for a microelectrode array exposed to 20 mM glucose with a corresponding Nyquist phase angle plot. For simplicity, only this concentration is shown, since similar profiles are seen for all of the glucose concentration ranges studied. Figure 11c shows a straight line profile of the real Z *versus* the imaginary Z components with a slope close to unity, indicative of a diffusion controlled reversible process, where the phase angle (θ) profiles seen within Figure 11b would appear to be dictated by the capacitance of the polymer film in conjunction with the double-layer contribution within the circuit. We have only considered how recorded impedance values, or components thereof vary under differing conditions, as opposed to how percentage changes in impedimetric behaviour vary, even though the total impedance measured may be dominated by the background impedance of the circuit. Percentage changes of microelectrode array glucose sensors across a range of glucose concentrations as a function of frequency under aerobic conditions are shown within Figure 11d. Percentage impedances are reported relative to the corresponding impedance at 0 mM glucose at a particular frequency, as shown in Figure 11d. These increases in impedance may be plotted with respect to the analyte concentration in the form of a calibration plot, Figure 11e, clearly demonstrating this approach for interrogation. The most prominent feature of this data is that impedance minima are observed at frequencies between 10 Hz and 100 Hz. Similar responses are seen under anaerobic conditions (not shown for clarity), although the percentage changes in response are not so pronounced [33].

We have already shown that the impedance values are modulated by enzyme catalytic behaviour, although the nature of the impedance changes have not yet been considered. At this stage the possible mechanisms by which the conductivity of the poly(aniline) may be modulated should be considered. Poly(aniline) has three differing forms [38]. The first mechanism by which glucose oxidase may give rise to redox modulated conductivities within the poly(aniline) involves the catalysed production of H_2_O_2_ [38]. Cooper and Hall [39], have shown that in aerobic conditions, H_2_O_2_ would be expected to play a major role in the modulation of the impedimetric behaviour of poly(aniline). The responses observed under anaerobic conditions could clearly not be accounted for by such a mechanism, since without a supply of molecular oxygen as an electron acceptor from the enzyme, H_2_O_2_ could not be produced. Since we have established that the impedimetric responses are due to enzyme catalytic behaviour, it follows that electron donation from the enzyme must be occurring by some other mechanism. Since the sensor responses under aerobic conditions are always greater than those observed under anaerobic conditions, it is therefore probable that the generation of H_2_O_2_ in aerobic conditions might contribute to this behaviour—even if additional mechanisms are involved in the overall response. A number of other possible mechanisms to explain anaerobic glucose oxidase responses have also been proposed by other workers. One of the most controversial of these is via a possible direct electron transfer occurring between the active site of enzyme and the polymer [39]. The distance through which electrons would have to traverse would be in excess of 1.3 nm (http://www-biol.paisley.ac.uk, academic website containing enzyme database) and would on first reflection appear to be prohibitive. Other workers have shown, however, that electron tunnelling can occur across distances that had previously been thought impossible via complex pathways of unsaturated and delocalised bonds together even with some saturated bonds and free space [40,41]. It should not be forgotten that gluconolactone may be produced under anodic conditions as long as a surrogate electron acceptor for the enzyme is provided. Gluconolactone readily hydrolyses to form gluconic acid and this may easily protonate the polymer allowing another route for altering the conductivity of the polymer. Skinner and Hall [42] have previously shown that gluconolactone produced under anaerobic conditions may give rise to conductivity changes within the polymer via interaction of gluconolactone with the emeraldine base to form a zwitterion complex that itself may be readily oxidised through to the perigraniline form of the polymer. While this behaviour would account for anaerobic impedimetric responses for our sensors to glucose, it would be difficult to fully explain why the response profiles we have observed exhibit minima at frequencies of approximately 10 Hz to 100 Hz. We believe that this behaviour could be linked to the hydrogen bonding of water to the imine centre of the polymer.

#### 3.2.2. Alcohol Oxidase

A number of alcohol oxidase-based enzyme electrodes containing positively charged polymers have previously been reported [43,44]. Alcohol oxidase was chosen as the most suitable oxidase enzyme for further study since a range of oxidases have previously been successfully immobilised within poly(aniline) [42]. Furthermore, the *Hansenula polymorpha* strain of alcohol oxidase has demonstrated stability at pH 5.5, and has moreover reported specificity toward primary alcohols (such as ethanol) which clearly has significance for both the alcoholic drinks industry as well as drink-driving legislation purposes, to quote but two examples. Poly(aniline) in its conductive form has a positive charge and composites of alcohol oxidase with a positively charged dextran derivative have shown enhanced stability over samples with no polymer [43]. For the polymerisation of alcohol oxidase microelectrode arrays, a 0.1 M aniline solution was prepared using the acetate buffer. Alcohol oxidase (500 U mL^−1^) was prepared in distilled water to avoid denaturing the enzyme. 2.7 mL of the buffer and enzyme preparation (1.35 mL each) were mixed in a small volume PTFE cell, immediately prior to the electrochemical coating procedure being performed. Aniline/AOD was polymerised from the acetate buffer solution onto a previously constructed microelectrode array by potentially sequentially cycling as previously described for Aniline/GOD protrusions. The working electrode was then immediately submerged in deionised water.

**Figure 12.**
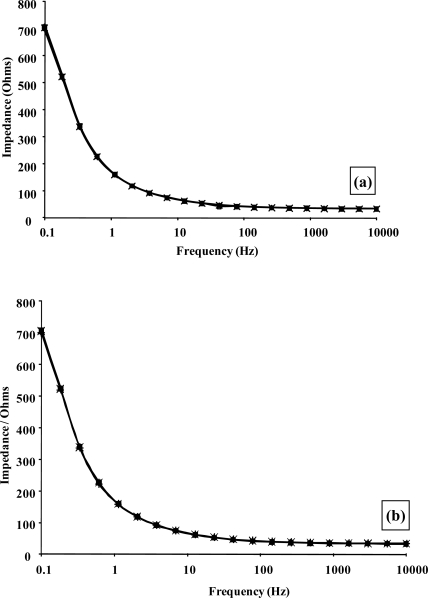

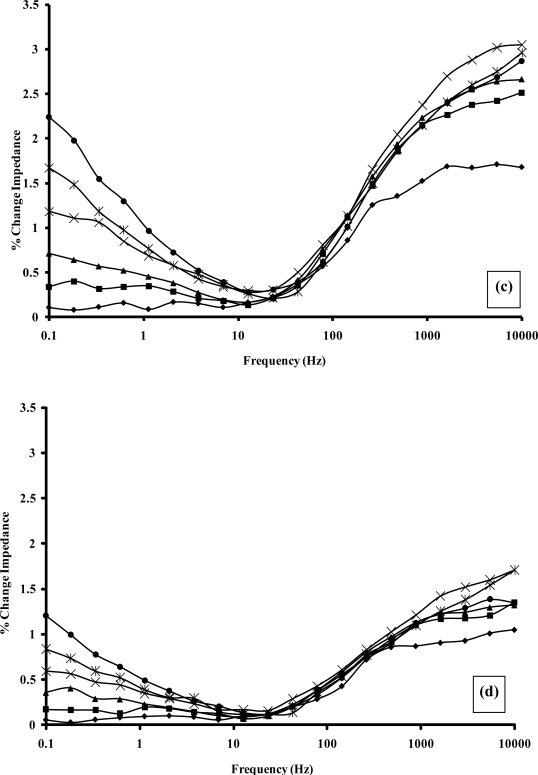
Plot of total impedance versus frequency for a poly(aniline)/alcohol oxidase microelectrode array exposed to a range of ethanol concentrations under **(a)** aerobic and **(b)** anaerobic conditions: (♦) 0 mM; (■) 1 mM; (▴) 5 mM; (×) 10 mM; (*) 20 mM; (●) 30 mM; (+) 40 mM—N.B. Plots are seen to converge due to scale utilised. Plots of percentage change (relative to 0 mM ethanol) impedance versus frequency for a poly(aniline)/alcohol oxidase microelectrode array exposed to a range of ethanol concentrations under **(c)** aerobic and **(d)** anaerobic conditions: (♦) 1 mM; (■) 5 mM; (▴) 10 mM; (×) 20 mM; (*) 30 mM; (●) 40 mM.

**Figure 13.**
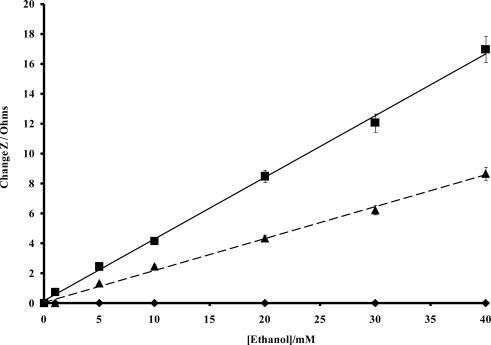
Calibration plot for impedance changes (at 0.1 Hz) for a poly(aniline)/alcohol oxidase microelectrode array *versus* ethanol concentration under; (a) aerobic (■) and (b) anaerobic (▴) conditions (c) control experiment with an enzyme free microelectrode array (♦).

In order to quantify the enzyme modulated resistance and capacitance changes within the polymer, impedance spectra were recorded for alcohol oxidase/poly(aniline) sonochemically fabricated microelectrode arrays across a range of alcohol concentrations using a frequency range 0.1–10 kHz, under both aerobic and anaerobic conditions, as shown within Figure 12a and 12b, respectively. In accordance with previous findings [45], significant decreases in impedance are observed as the frequency increases, and this masks the changes that are obtained with differing concentrations of ethanol when the data is plotted in this format. For this reason, percentage impedance changes were again plotted as a function of frequency for differing concentrations of ethanol (Figure 12c and 12d), under aerobic and anaerobic conditions respectively, in a similar manner to the way in which enzymic responses to glucose were previously presented by ourselves. It is immediately obvious from the data in Figure 12 that a broadly similar trend in behaviour is seen for both the aerobic and anaerobic alcohol oxidase/poly(aniline) system as for the glucose oxidase/poly(aniline) system. The responses observed for the anaerobic system are again smaller than those observed under aerobic conditions. Control impedance spectra for enzyme-free microelectrode sensors, are again not shown for brevity, but as shown in the calibration profile (Figure 13), fail to show any response to ethanol and in this way demonstrate that the impedimetric changes observed are indeed enzymic in origin. Data from Figure 12 may be used to form calibration profiles (Figure 13), if the impedance changes are plotted as a function of concentration at 0.1 Hz in accordance with previous studies [45]. The r^2^ values are 0.998 (aerobic) and 0.996 (anaerobic). The nature of the impedance responses cannot be attributed solely to the effects of hydrogen peroxide, since enzymic responses are observed under both aerobic and anaerobic conditions, and it is clear that peroxide may only be produced by oxidase enzymes in the presence of oxygen.

**Figure 14.**
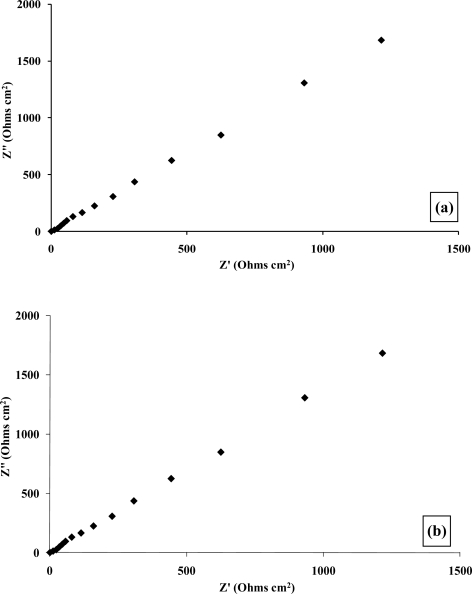

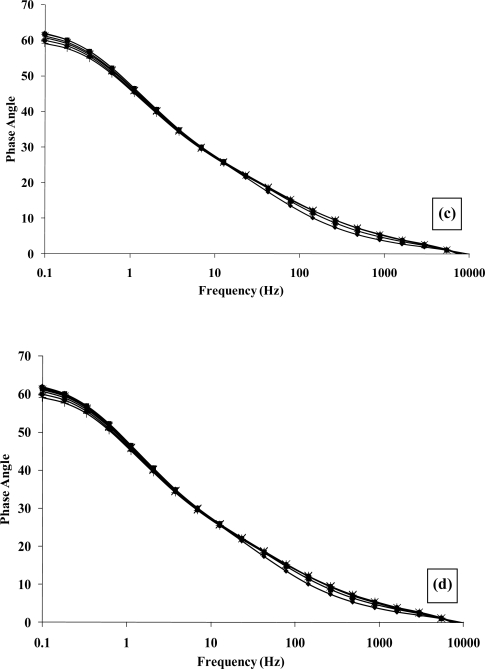
Plot of real *versus* imaginary impedance for a poly(aniline)/alcohol oxidase microelectrode array exposed to ethanol (40 mM) under **(a)** aerobic and **(b)**, anaerobic conditions. Plot of phase angle *versus* frequency for a poly(aniline)/alcohol oxidase microelectrode array exposed to ethanol (40 mM) under **(c)** aerobic and **(d)** anaerobic conditions: (♦) 0 mM; (■) 1 mM; (▴) 5 mM; (×) 10 mM; (*) 20 mM; (●) 30 mM; (+) 40 mM.

Complex plane impedance plots of Z′ *versus* Z″ under aerobic and anaerobic conditions, Figures 14a and 14b respectively, are broadly similar to those observed for the glucose oxidase system [45]. The plots of the phase angle θ, with respect to ethanol concentration under aerobic and anaerobic conditions, Figures 14c and 14d respectively, again demonstrate the capacitive contribution to the cell as previously described. Our findings demonstrate that enzyme modulated impedance changes may also be seen for alcohol oxidase/poly(aniline), albeit at a smaller magnitude than for the glucose oxidase/poly(aniline) system. It has been proposed in earlier work [42] that the impedimetric response within the glucose oxidase/poly(aniline) system may be attributed to the nucleophilic attack of poly(aniline) on gluconolactone to give a zwitterionic reaction product.

It was also reported [42] that an alcohol oxidase/poly(aniline) system did not show any impedimetric response and therefore by inference an absence of zwitterion polymer protonation effects. Alcohol oxidase under aerobic conditions, catalyses the production of acetaldehyde and H_2_O_2_ according to Equation (5) in the presence of ethanol:

(5) $$\text{ethanol}+O_{2}\overset{\text{Alcohol}\;\text{Oxidase}}{\to }\;\;\;\text{acetaldehyde}+H_{2}O_{2}$$

**Figure 15.**
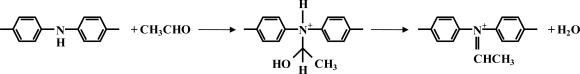
Suggested reaction of acetaldehyde with poly(aniline).

Acetaldehyde could also react with poly(aniline) to give a variety of reaction products such as a carbinol amine or a Schiff base type structure as shown in Figure 15 [46]. Products of this type however will be much more susceptible to hydrolysis back to the starting materials, in comparison to the glucose oxidase/poly(aniline) system. In turn, it might be expected that a smaller extent of protonation of the emeraldine base occurs and this in turn gives rise to the smaller responses observed for this system.

Due to the lowered response, it is possible that this effect may not have been detected by planar electrodes used by other workers, and that our responses have by contrast been enhanced by the hemispherical diffusional mass transport characteristics and enhanced sensitivity that our sonochemically fabricated microelectrode systems have been shown to exhibit. It has also been proposed earlier [45] that the minima impedance change observed at frequencies of approximately 10–100 Hz may be related to stabilisation of H-bonded water to the imine centre of the polymer. Some evidence is again seen for a minima in percentage impedimetric response, and is once more observed at frequencies ranging between approximately 10 and 100 Hz for the alcohol oxidase/poly(aniline) system studied here (Figure 12c and 12d), although it should be acknowledged that these effects are smaller than those observed for the glucose oxidase/poly(aniline) system. Anaerobic responses are again smaller than those observed under aerobic conditions [33].

This section details the amperometric and impedimetric investigations of sonochemically fabricated microelectrode arrays developed upon oxidase-based systems. Here, the investigations into the use of microelectrode array sensors, modified with poly(aniline) protrusions, containing a biological entity (in this instance oxidase enzymes) are detailed. We have shown that both glucose and alcohol oxidase may be successfully “entrapped” within poly(aniline), grown from the ultrasonically ablated pores. The amperometric response of these modified sensors were then measured over a range of glucose and ethanol concentrations respectively and the resulting calibration profiles showed that an amperometric enzymatic sensors were produced that could be exploited for a variety of applications in which stir-independent responses are necessitated.

The electrodes were exposed to differing concentrations of glucose or ethanol and calibration profiles recorded under both aerobic and anaerobic conditions; this clearly demonstrated that responses could again be observed in both environments. Impedimetric responses of the both the glucose oxidase/poly(aniline) and alcohol oxidase/poly(aniline) microelectrodes were compared to those of a blank (enzyme-free) systems. The resulting spectra clearly demonstrated that in both instances the observed responses were indeed enzymic in nature. Although in the case of glucose oxidase-based systems the higher signal responses within aerobic conditions can be attributed to the increased production of H_2_O_2_ in this instance, we have also suggested a number of possible mechanisms that yielded the signal responses in anaerobic conditions (as well as contributing towards aerobic responses), such as electron-tunnelling or hydrogen bonding effects. In the case of alcohol oxidase-based systems, our findings lend further support to the suggestion made previously by us that the impedimetric response may be due to the effect of a zwitterion complex formed between the carbonyl enzymic product and the polymer in addition to effects associated with the dissociation of water hydrogen bonded to the imine centre of the poly(aniline). Previous groups have to date failed to observe these effects with the alcohol oxidase system [42] and we believe that the use of microelectrodes and the assisted diffusional mass transport associated with the hemispherical diffusional profiles they experience has allowed us to observe the smaller signals associated with this enzyme.

### 3.3. Poly(siloxane) Coatings

One problem with sensor systems is a loss of signal linearity at high analyte concentrations (>10 mM). We describe here the modification of a sonochemically fabricated microelectrode glucose oxidase enzyme array via the deposition of a poly(siloxane) coating for the improvement of signal linearity.

**Figure 16.**
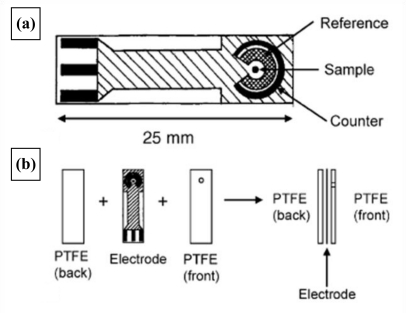
Design of the ceramic electrodes **(a)** and PTFE masks used during poly(siloxane) deposition **(b)**.

The fabrication of microarrays with poly(aniline) protrusions were fabricated as described previously (section 2.1). Dimethyldichlorosilane, 0.5 mL, was placed in a watch glass at the bottom of a sealed reaction vessel kept at atmospheric humidity. A stage was designed to allow for the deposition of the poly(siloxane) directly at the working electrode surface (Figure 16) thereby ensuring that the counter and reference electrodes, and all the electrode contacts were not exposed to the silane vapour. The electrode was then suspended 5 cm above the dichlorodimethylsilane liquid for pre-determined periods. The stage was then removed from the vessel and the electrode stored. Previous work within this group has been focused towards the development and exploitation of ultra-thin film composite membrane technology for the construction of microelectrodes. A technique for depositing poly(dimethylsiloxane) from dichlorodimethylsilane has been previously shown by us to give thin films which offer low diffusional resistance to glucose but which minimise surface biofouling [47], thus offering protection against electrode passivation effects, and therefore allowing for the linearisation of sensor responses. Permittivity coefficients were measured for these films [47] and showed a much higher permeability for oxygen than for glucose; this is important since oxygen is a co-factor in the glucose–glucose oxidase reaction and we do not wish it to become depleted at the electrode surface and cause the sensor to reach saturation.

**Figure 17.**
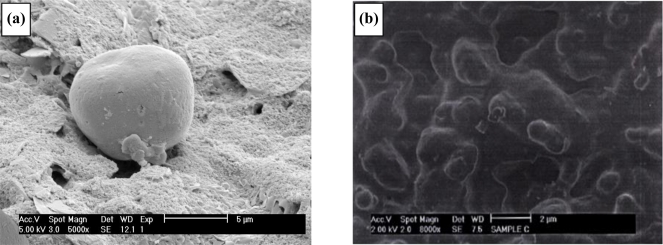
SEM of poly(aniline) enzyme containing polymer protrusion microelectrode arrays **(a)** before and **(b)** following coating with poly(siloxane).

**Figure 18.**
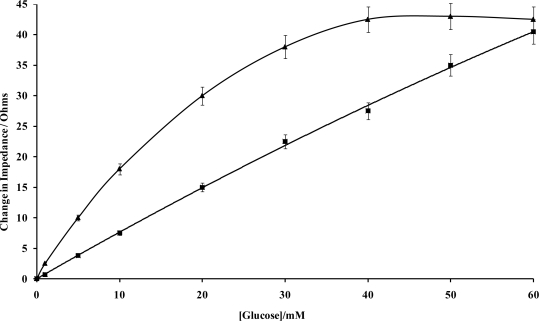
AC impedance calibration plot for glucose oxidase microelectrode arrays: uncoated (▴), with poly(siloxane) coating (■).

An SEM of the working electrode (Figure 17a) clearly shows one of the resulting polymer/enzyme protrusions. SEM images of the siloxane-coated enzyme microelectrodes are shown in Figure 17b. A film can be seen to cover the entire electrode surface. In order to assess the performance of the ultra-thin film as a coating for the linearisation of sensor responses, the AC response to glucose of an enzyme microelectrode array/ultra-thin polymer film was compared to that of a similar electrode lacking an ultra-thin polymer film coating. AC impedimetric responses were determined over a range of frequencies from 0.1 Hz to 10 kHz. Again the responses were verified to be free of stirring (enforced convectional) effects. The Bode and Nyquist plots were almost identical to those published previously [45] and therefore are not shown for brevity. Earlier work showed that the greatest variations in impedance occurred at 0.1 Hz [45] and similar results were obtained for the siloxane-coated membranes. When the differences in impedance at frequency of 0.1 Hz are plotted for microelectrodes exposed to varying glucose concentrations in pH 7.4 buffer, a calibration plot is obtained (Figure 18). The instrumentation required for commercial exploitation of biosensors may be greatly simplified if the sensor gives a linear response with respect to analyte concentration. The resulting calibration graph (Figure 18) shows that the siloxane coating lowers the magnitude of sensor response compared to uncoated microelectrodes whilst also linearising the sensor responses between 0 and 60 mM glucose (r^2^ = 0.671 (uncoated), r^2^ = 0.995 (silane coated)). We have determined the reproducibility of sensor responses to offer <5% variability [45].

We have combined two disparate biosensor technologies to form a novel enzyme-based electrochemical sensor. Initially we described the examination of a commercially available ceramic based disposable electrode as a suitable host for the fabrication of a microelectrode array. Microelectrode arrays were fabricated as described previously via sonochemical ablation; these arrays again displayed true stir-independent responses. Enzyme/polymer protrusions were then again electrochemically deposited [45] at the host microelectrode templates so as to produce an enzyme (glucose oxidase) ultra-microelectrode array.

Enzyme microelectrode arrays were then coated with an ultra-thin film coating of poly(siloxane) (silicone) so as to provide a covering substrate diffusion limiting layer for the linearization of sensor responses. This coating has been shown to extend the useful analytical concentration range for the sensor via linearisation of its response.

### 3.4. Organophosphate Pesticide Detection Using Microarrays

Modern agricultural practices often involve the use of organophosphate pesticides to control insect infestation. However increasing concern is being shown towards their indiscriminate use due to the potential deleterious immediate and long term effects they may cause to the environment, livestock and human health [48,49]. Organophosphate pesticides (OPs) appear to have taken the place of many of the organochlorine pesticides previously used due to their lower persistence in the environment whilst still remaining effective. Unfortunately OPs are neurotoxins and therefore present a serious risk to human health. The possibility of OP contamination of our food and water supplies necessitates the development of analytical methods for the reliable detection of pesticides, to safeguard both food supplies and the environment. Possible contamination of water supplies is a hazard and legislation has now been passed (European Commission: EU Water Framework Directive 2000/60/EC, European Commission: Drinking Water Directive 98/83/EC, which recommends maximum levels within water supplies of 0.1 mg L^−1^ for individual pesticides and 0.5 mg L^−1^ for total pesticide) to restrict pesticides within water supplies. Due to concerns about these materials it appears probable that these permitted levels will decrease. More recently, along with concerns about accidental release there is also the possibility of deliberate use of these nerve agents, as happened for example, with the Sarin gas attack on the Tokyo underground in 1995.

Current methods for determination and/or the monitoring of pesticides include gas and liquid chromatography and various spectroscopic techniques [50]. Many of these methods are time consuming, not sufficiently sensitive and/or require complex sample preparation and are not suitable for continuous monitoring. The development of a simplified analytical approach would prove highly beneficial.

Biosensor technology has been utilised as a potential solution to this problem, especially electrochemical biosensing techniques since they are amongst the easiest and most inexpensive methods for detection of binding events. Many groups have previously demonstrated the fabrication of biosensors for the detection of pesticides [51]. Usually these do not directly detect the pesticides itself. Instead it is known that pesticides, even at very low concentrations, can inhibit enzyme reactions, usually by “poisoning” of the enzyme. It is possible to first construct an enzyme-electrode, measure its response when exposed to a suitable concentration of its substrate and then expose it to a dilute pesticide solution. Further exposure to the initial substrate concentration and comparison with the response prior to pesticide exposure allows quantification of the inhibition which can then be correlated with pesticide concentration.

Cholinesterases, such as acetyl cholinesterase (AChE) catalyse the hydrolysis of choline esters to the corresponding carboxylic acid and choline, Equation 6.

(6) $$\text{Acetylcholine}+H_{2}O\overset{\text{Acetyl}\;\text{Cholinesterase}}{\to }\;\;\text{Choline}+\text{Acetic}\;\text{Acid}$$

The inhibition of this reaction has been used for the detection of organophosphate and other pesticides due to its high specificity and sensitivity [51]. Usually the simple amperometric detection of the product of the enzyme catalysed ester hydrolysis reaction is utilised [52]. Screen-printed carbon electrodes, often containing electrochemical catalysts, are the favoured substrate for these sensors due to their inexpensiveness and ease of mass-production. Enzymes can be immobilised onto these substrates using a wide variety of methods. A discussion of the field of pesticide detection using biosensors is outside the scope of this paper but is reviewed here [51].

#### 3.4.1. Application of Microelectrodes

**Figure 19.**
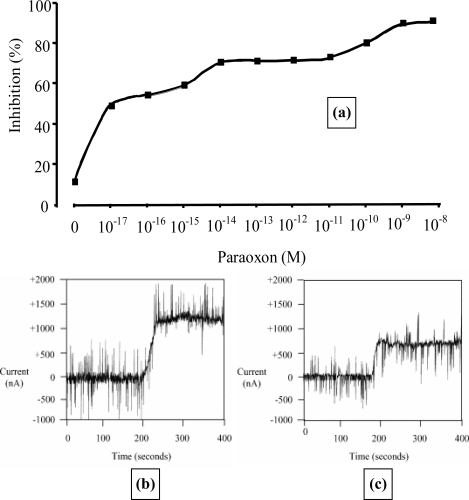
Calibration curve **(a)** for paraoxon inhibition of acetylcholinesterase-modified CoPC electrodes for paraoxon concentrations between 10^−8^ and 10^−17^ mol L^−1^. Insets show a typical current transient response for an AChE-modified electrode to 2 mmol L^−1^ acetylthiocholine chloride, before **(b)** and after **(c)** the addition of 1 × 10^−17^ mol L^−1^ paraoxon.

Microelectrode arrays have also been used for the detection of organophosphates [51,53,54]. Acetylcholine esterase hydrolyses acetylthiocholine chloride to give thiocholine which can be easily detected using screen-printed carbon electrodes doped with cobalt phthalocyanine (CoPC) [53,54]. The use of the CoPC catalyst reduces the working potential to approximately +100 mV (*versus* Ag/AgCl), thereby minimising interference from other electroactive compounds. We therefore utilised our sonochemical fabrication technique to form micropore arrays from which microarrays of poly(aniline) containing entrapped acetylcholine esterases were grown [55]. A genetically modified AChE, designed to maximise sensitivity was incorporated, as was an I^125^ labelled AChE to quantify the amount of enzyme deposited, which in this instance corresponded to 0.15 units activity [53]. Measuring the amperometric response of the electrode in 2 mmol L^−1^ acetylthiocholine before and following exposure to paraoxon solutions of various concentrations allowed calibration profiles of the inhibition of enzyme activity against pesticide concentration to be constructed (Figure 19a). A typical current transient response for an AChE-modified electrode to 2 mmol L^−1^ acetylthiocholine chloride is shown in Figure 19b. Levels as low as 10^−17^ mol L^−1^ paraoxon could be reproducibly detected and a current transient response after exposure to this ultra-low concentration is shown (Figure 19c). Other workers have also reported pesticide detection at comparable levels using acetylcholine esterase immobilised on microporous conductive carbon [56].

**Figure 20.**
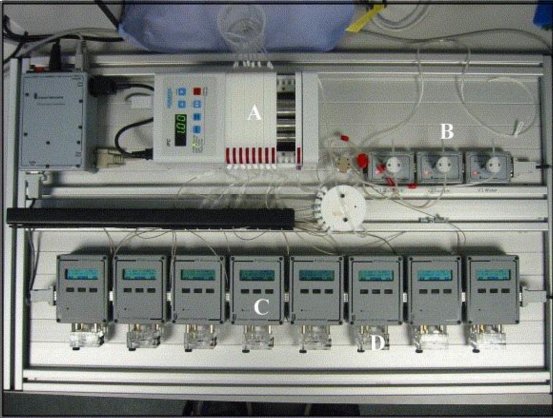
Flow-injection analyser. **(A)** Pump set at 1 mL min^−1^, **(B)** injection valves for substrate and pesticide samples, **(C)** one of eight potentiostats, and **(D)** flow cell which comprises one sensor.

These AChE containing micro-electrode arrays could also be utilised within a flow injection system [52]. A sample was separated and flushed simultaneously through eight cells, each containing a screen-printed electrode and fitted with a separate bespoke mini-potentiostat (Figure 20). This offers the possibility of allowing multiple measurements to be made on a single water sample. Each electrode could be designed to be specific for a different pesticide due to the availability of different AChE mutants. Alternatively native enzymes could also be installed as controls to show the presence of other interfering pollutants such as heavy metals. These arrays again proved successful with a high sensitivity for pesticide detection, limits of detection for dichlorvos as low as 1 × 10^−17^ mol L^−1^ and parathion and azinphos both at concentrations as low as 1 × 10^−16^ mol L^−1^ [54].

We have developed sensitive and selective enzyme electrodes based on poly(aniline) microarrays capable of measuring concentrations of several pesticides down to levels hitherto undetectable (1 × 10^−17^ mol L^−1^). There is the potential for the use of multiple electrodes, pattern recognition software and flow injection techniques to enable determination of different pesticides from mixtures as well as subtraction of matrix effects such as heavy metals from the system. The automation within this system allows use of the instrumentation by unskilled personal, thereby removing the sensing platform from specialized laboratories and making it directly available to the end-users. The relative low cost of electrochemical technology and mass produced screen printed electrodes (which can be utilised as single shot sensors) makes it an attractive, inexpensive alternative to other technologies. This low cost, coupled to equipment portability, means it could conceivably be utilised in the field as well as under laboratory conditions.

### 3.5. Immunosensors Based on Poly(aniline) Microarrays

The interaction of antibodies and antigens leads to formation of tightly bound complexes with a very high degree of both sensitivity and selectivity. This selectivity has led to the development of such widely used assays such as the Enzyme Linked Immunosensor Assay (ELISA). There has been a wide interest in exploiting the selectivity of antibody-antigen interaction within electrochemical based immunosensors. The development of immunosensors which give electrochemical responses is a widely studied topic as detailed in several recent reviews [57–59].

Our group has also pioneered the development of sonochemically fabricated microarrays of conductive polymers for use in enzyme based sensors and also the studied the development of immunosensors which utilise either polymer entrapment or an affinity based protocol. Our first venture into this field involved the development of immunosensors for bovine serum albumin (BSA) and digoxin. Poly(pyrrole) was laid down on a carbon electrode by simple electrochemical polymerisation in solutions that contained antibodies to either BSA or digoxin. Studies using a radioactive labelled antibody showed that up to 2–3 μg of antibody may be successfully entrapped into the conducting poly(pyrrole) films [60].

Unfortunately the interaction between antibody and antigen does not release any electrochemically active species as observed for the enzyme reactions, so simple voltammetry will not measure a response upon binding. Instead we utilised an AC impedance protocol [61] and showed that the binding of antigen gave measurable changes in the impedance, allowing the development of immunosensors for digoxin and BSA. However problems exist with this technique. The first complication is with the deposition of a conductive polymer film which may require conditions such as a low pH under which the biological species of interest may suffer some loss of activity or even be completely denatured. Also entrapped antibodies may experience steric effects from the matrix or be wrongly orientated to bind the antigen, especially in the case of bulky antigens such as proteins. Therefore in later work we utilised a method in which poly(aniline) coated screen-printed carbon electrodes were used as substrates for antibody immobilisation via avidin-biotin interactions.

**Figure 21.**
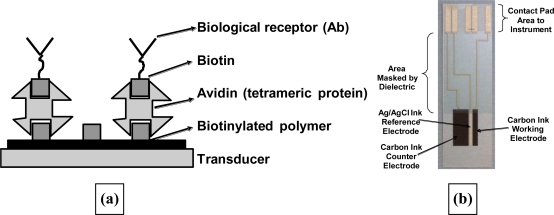
**(a)** Schematic of the immobilisation of antibodies on screen-printed electrodes **(b)** screen printed electrodes used within this work.

High quality poly(aniline) films could be deposited at low pH and then biotinylated using commercial biotinylation reactions. The interaction between biotin units and avidin proteins is well known as a method of selective immobilisation of various species. Therefore treating the biotinylated poly(aniline) with a solution of neutravidin allowed the deposition of a monolayer of the protein onto the conductive polymer. Meanwhile in a separate procedure, the same biotinylation reagent is used to treat antibodies to generate antibodies with a number, usually between two and four, of biotin units per antibody on average. Exposure of the avidin coated poly(aniline) electrode to these moieties caused deposition of orientated antibodies on the electrode surface as shown schematically in Figure 21.

We successfully utilised this approach to construct immunosensors for the antibiotic ciprofloxacin which could detect levels of 1 ng mL^−1^ of the antibiotic, not only in PBS [62] but also in milk [63]. We have also developed electrochemical immunosensors for myelin basic protein—a marker for stroke and multiple sclerosis [64] and Internalin B, a surface protein marker for Listeria [65].

Different methods of immobilisation were also investigated, a programme of work which is described in more detail elsewhere [66]. Initial studies had utilised antibodies immobilised in planar polymer films but our work on the affinity modified planar electrodes gave higher sensitivities than noted for these films. It was therefore decided to directly compare entrapped and affinity immobilised immunosensors and also to compare planar electrodes with microelectrodes. Immunosensors containing anti-digoxin were fabricated by utilising the neutravidin-biotin procedure and shown to have limits of detection approximately 1,000 times less [66] than electrodes where the antibody was simply entrapped within a polymer film. Similarly entrapment of BSA in microelectrode arrays led to almost a 10,000 fold lowering in detection limit compared to entrapment within planar films.

One shortcoming of immunosensors is that they effectively measure deposition of material on the electrode surface. However many materials, either the antigen itself or interfering species found especially in complex media like blood can undergo non-specific interactions with surfaces. This can interfere with immunosensor performance, leading to erroneously elevated results. In an attempt to correct for non specific interactions, identical sets of immunosensors were therefore fabricated containing a non-specific IgG antibody (AIgG) in place of the specific antibodies. Results for these electrodes were obtained in exactly the same manner as for the specific electrodes. Therefore within this work we will only detail results which have been corrected. To obtain a “corrected” calibration profile, for an APSA immunosensor, non-specific responses were subtracted from the specific responses over the entire analytical concentration range.

Since both the affinity protocol and the use of microelectrodes led to increases in sensitivity, these two techniques were combined. This study was performed using antibodies for prostate specific antigen (PSA). Prostate cancer is a disease most frequently encountered in men over fifty and second only to lung cancer for the number of male deaths in the US, 30,350 deaths in 2005 [67] and the UK, 10,000 deaths in 2005 [68]. Levels of a 34 kD glycoprotein PSA, which is manufactured almost exclusively by the prostate gland are often (but not always) elevated in the presence of prostate cancer and other prostate disorders [69]. We will only give an overview of the construction and utilisation of microarray based biosensors for PSA but a much more detailed description has been published [70].

As before, poly(aniline) microarrays were fabricated using the methods described. In some cases, antibodies to PSA (APSA) were entrapped by growing the microelectrodes from solutions containing APSA. Alternatively microarrays of poly(aniline) were treated with the biotinylating agent, followed by avidin and finally biotinylated APSA to give microarrays with immobilised antibodies. Impedance analyses were performed from 1 Hz to 10,000 Hz (±5 mV amplitude perturbation) offset to the formal potential of the redox probe (+0.12 V *versus* Ag/AgCl). A redox mediator mixture, 5 mmol L^−1^ each of [Fe(CN)_6_]^3−/4−^, was utilised to enable the performance of faradaic impedance spectroscopy. Baseline traces were obtained for the APSA doped sensors in phosphate buffer containing the mediator but no antigen—these were subsequently exposed to a range of concentrations from 1 to 300 ng or pg mL^−1^ PSA in pH 7.4 phosphate buffer containing [Fe(CN)_6_] ^3−/4−^.

**Figure 22.**
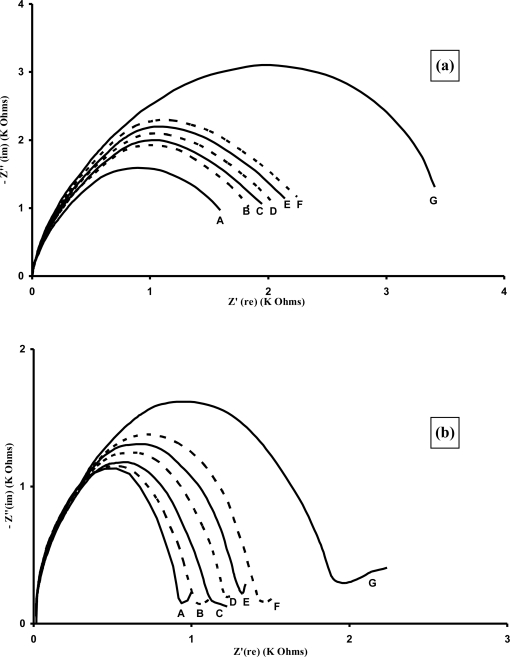
Specific binding of PSA for **(a)** entrapped APSA, Nyquist plot, concentrations of PSA (A) 0; (B) 1; (C) 2; (D) 3; (E) 4; (F) 5 and (G) 10 ng mL^−1^; **(b)** affinity immobilised APSA, Nyquist plot, concentrations of PSA (A) 0; (B) 1; (C) 2; (D) 3; (E) 4; (F) 5 and (G) 10 pg mL^−1^.

A series of typical Nyquist plots (Figure 22) are shown for the sensors containing (a) affinity immobilised APSA and (b) entrapped APSA. The immobilised sensors display large changes in the Nyquist plots at much lower concentrations of antigen than occurred for the entrapped microelectrode arrays. It appears that the effects of affinity binding and the use of microelectrodes are complimentary and combining them leads to further enhancements in sensitivity and lowering of detection limits.

The semicircular shape of the Nyquist plots is characteristic of a surface modified electrode system where the electron transfer is slow and the impedance is controlled by the interfacial electron transfer [71]. From these curves it is possible to extract the surface electron transfer resistance. Relative electron transfer resistance changes from the baseline response can be plotted against the antigen concentration to give calibration profiles. Binding of antigen appears to hinder the access of the redox probe to the surface, thereby insulating it and increasing the electron transfer resistance. Entrapped APSA immunosensors gave linear responses to the analyte from 1 ng mL^−1^ to 200 ng mL^−1^ PSA and then reached a plateau. Affinity based immunosensors gave a linear response from 1 pg mL^−1^ to 100 pg mL^−1^ PSA.

**Figure 23.**
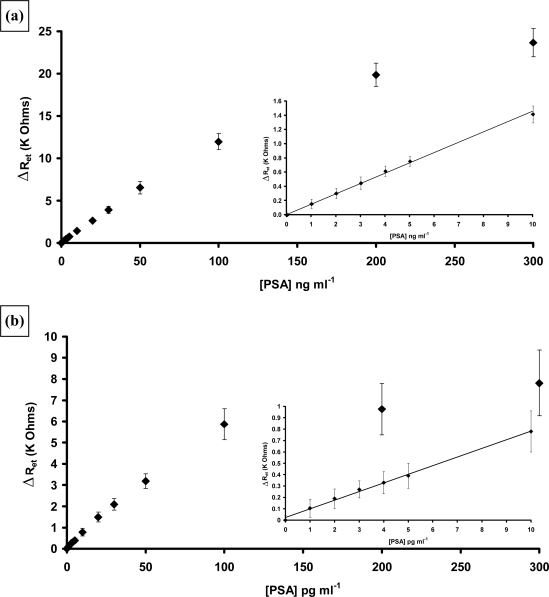
Corrected calibration plots showing changes in electron transfer resistance *versus* PSA concentration: **(a)** entrapped APSA 0–300 ng mL^−1^ PSA, inset 0–10 ng mL^−1^ PSA **(b)** affinity immobilised APSA 0–300 pg mL^−1^ PSA, inset 0–10 pg mL^−1^ PSA.

Measurements of the AIgG electrodes also gave changes in Nyquist curves and calibration plots could also be drawn, indicating that about 20% of the increase in electron transfer resistance was in fact due to non-specific effects [70]. Therefore these results were subtracted from the specific binding results to give the corrected results (average of 10 specific and 10 non-specific electrodes) as shown in Figure 23. As can be seen the immobilised immunosensors display much higher sensitivities and lower detection limits, with a linear range from 1 to 100 pg mL^−1^ PSA and the entrapped immunosensors from 1–100 ng mL^−1^ PSA. Insets in the figures show expanded plots for the lower concentrations.

It appears that the best sensitivities can be obtained by combining poly(aniline) microarrays with affinity based attachment protocols. The detection limit for PSA (three times the standard deviation of the baseline value) was 1 pg mL^−1^. It was therefore decided to study a range of other antigens. Two which were of interest were Neuron Specific Enolase (NSE), a 78 KDa protein and S100[β], a 21 KDa protein. Both of these proteins, when found in blood, are markers for transient ischemic events such as strokes. Stroke is the third leading cause of death in the USA and the UK after heart disease and all cancers [72,73]. Delays of above three hours after stroke onset in medical intervention may contribute to patient deterioration and disability. To help identify those individuals at risk, there is a need for a rapid, sensitive and specific diagnostic assay for stroke events. Both NSE and S100[β] concentrations in blood are elevated after stroke or other cerebral injury. We therefore instigated a study into the detection of stroke marker proteins. Again detailed descriptions of this work have been published [74,75] so an overview will be given.

**Figure 24.**
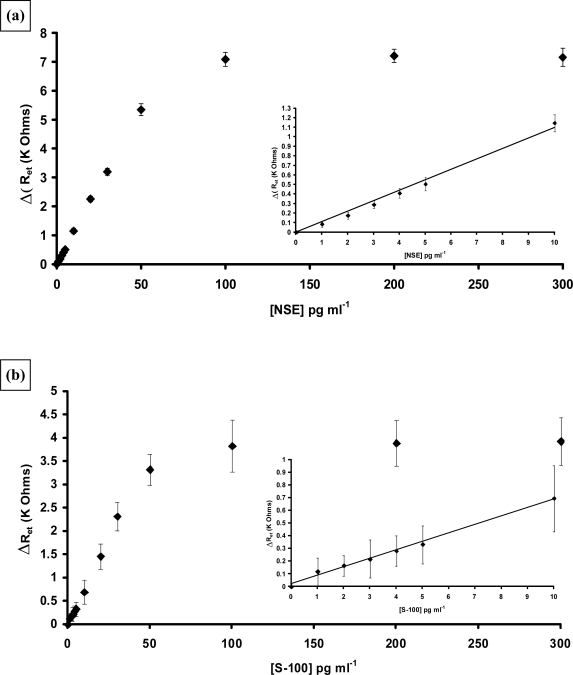
Corrected calibration plots showing changes in electron transfer resistance *versus* antigen concentration: **(a)** affinity immobilised ANSE 0–300 pg mL^−1^ NSE inset 0–10 pg mL^−1^ NSE **(b)** affinity immobilised AS100[β] 0–300 pg mL^−1^ S100[β], inset 0–10 pg mL^−1^ S100[β].

Antibodies to NSE and S100[β] were biotinylated and immobilised at the surface of poly(aniline) microarrays as described for PSA. The analysis was carried out as before and strong specific responses noted for both proteins. Non-specific binding was also observed for both proteins, of a similar level to that noted for PSA and was therefore subtracted. Corrected calibration plots for both proteins are shown in Figure 24. Both sensors were capable of detecting low levels of the requisite antigen. For NSE a linear relationship was observed between concentrations of 1–50 pg mL^−1^ (Figure 24a) and the response tends towards a plateau as the sensor approaches saturation. A limit of detection for the immunosensor (three times the standard deviation of the baseline value) of 0.5 pg mL^−1^ is obtained. The sensitivity of the S100[β] is somewhat lower than the other two immunosensors as shown in Figure 24b, however it still gives good response at extremely low levels of antigen with the limit of detection of the AS100 immunosensor (three times the standard deviation of the baseline value) being 1 pg mL^−1^ S-100[β].

Attempts were made to measure the effect on other proteins in the samples and whether they would interfere. Affinity bound immunosensors for all three antigens were fabricated and the testing protocol repeated except that in each case the solution, besides containing the antigen also contained a cocktail of 10 ng mL^−1^ each of several interfering proteins (in the case of PSA these were BSA, HSA, CA125 protein (tumour marker), S100[β] and NSE) along with the antigen. With the other two antigens, PSA 10 ng mL^−1^ replaced the antigen in the cocktail. For all three antigens the mean “corrected” electron transfer changes showed a variation of less than 5% from the unadulterated samples. This indicates either minimal adsorption of potential interfering proteins or if it does occur it does not significantly affect the biorecognition events (stable immunocomplex formation) and its effect on impedance.

**Figure 25.**
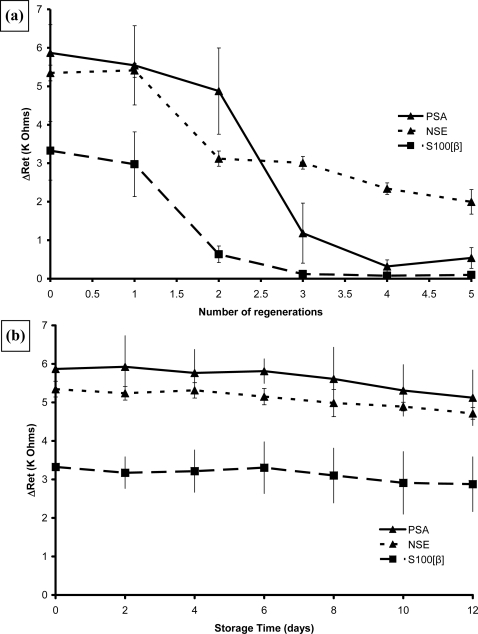
Effect of **(a)** regeneration and **(b)** storage on the mean change in electron transfer resistance at 100 pg mL^−1^ antigen for affinity-immobilised immunosensors.

The regeneration and stability of these affinity bound complexes were also tested. Samples of all three immunosensors were tested over the range of concentrations shown as before, given three 1 minute washes in 0.1 mol L^−1^ HCl and then retested. Figure 25a shows the overall results for the response at 100 pg mL^−1^ antigen for the immunosensors. As can be seen the PSA sensors could be rinsed and reused once with no loss of response, a second wash lowered it to 85% and further washes caused further loss of performance. Similar behaviour was observed for NSE and S100[β] in that the first regeneration did not affect them but after a second washing the performance had dropped to 60% and 15% of the original respectively. Washing immunosensors containing embedded APSA with 0.1 mol L^−1^ HCl completely destroyed their activity.

Samples of all three immunosensors were stored dry at 4 °C for up to twelve weeks and two of each tested at two week intervals. As Figure 25b shows, the performance did not deteriorate with storage. Samples containing embedded PSA were also tested, and were found to be stable for up to six weeks but after 12 weeks their performance had deteriorated to about a third of “fresh” sensors [66].

This section provides an overview towards the development of a viable approach towards the labeless sensing of markers for cancer and stroke, thus demonstrating the generic nature of the technology. The combination of microarrays and affinity binding protocols have given sensors with good linear ranges and limits of detection as low as 1 pg mL^−1^ for PSA, NSE and S-100[β].

The immunosensors were reversible for one regeneration attempt although after this further acid treatment reduces the performance, probably due to either loss of or denaturation of the immobilised antibody. Negligible loss of sensitivity after storage for twelve weeks at 4 °C in the dry state was observed. Minimal interference was observed upon exposure to relatively high levels of other proteins, encouraging for possible future applications.

These initial studies show great promise and it should be noted the limits of detection of the affinity based sensors are far below the levels found in clinical samples. This high sensitivity means that clinical samples can be highly diluted in PBS before measurement, thereby greatly reducing the amount of initial sample required, simplifying sample handling and minimising the effects of potential interferents.
